# Detection of Abrin by Electrochemiluminescence Biosensor Based on Screen Printed Electrode

**DOI:** 10.3390/s18020357

**Published:** 2018-01-26

**Authors:** Shuai Liu, Zhaoyang Tong, Xihui Mu, Bing Liu, Bin Du, Zhiwei Liu, Chuan Gao

**Affiliations:** State Key Laboratory of NBC Protection for Civilian, Beijing 102205, China; 15155922415@163.com (S.L.); mxh0511@sohu.com (X.M.); lbfhyjy@sohu.com (B.L.); dubin51979@163.com (B.D.); liuzhw07@lzu.edu.cn (Z.L.); g.ch.chuan@263.net (C.G.)

**Keywords:** screen printed electrode, electrochemiluminescence, portable sensor, abrin

## Abstract

For the convenience of fast measurement in the outdoor environment, a portable electrochemiluminescence biosensor with the screen-printed electrode as the reaction center was developed, which possesses the characteristics of high sensitivity, small scale, simplified operation and so on, and has been used for in situ detection of abrin. First, combining with magnetic separation technique, the “biotin-avidin” method was used to immobilize the polyclonal antibody (pcAb) on the magnetic microspheres surface as the capture probe. Secondly, the Ru(bpy)_3_^2+^-labeled monoclonal antibody (mcAb) was used as the specific electrochemiluminescence signal probe. Then, the “mcAb-toxin-pcAb” sandwich model was built to actualize the quantitative detection of abrin on the surface of the screen-printed electrode. The linear detection range was 0.5–1000 ng/mL; the regression equation was Y = 89.251lgX + 104.978 (R = 0.9989, *n* = 7, *p* < 0.0001); and the limit of detection (LOD) was 0.1 ng/mL. The sensing system showed high sensitivity, excellent specificity and good anti-interference ability, and could be used for the analysis of trace abrin in various environmental samples with good recovery and reproducibility. Compared with the traditional electrochemiluminescence sensing device, its miniaturization and portability gives it potential to satisfy the requirement of in situ detection.

## 1. Introduction

Abrin from *Abrus precatorius* is a highly toxic ribosome inactivating protein. It has similar characteristics to ricin, which has been used as a terrorist threat, but more toxic [1,2]. As a category B agent of potential bioterrorism risk by the “Centers for Disease Control and Prevention Moran” [3], abrin’s detection and prevention has become a focus of the public security field. Currently, the methods to detect abrin mainly include enzyme-linked immunosorbent assay (ELISA) [4,5], radioimmunoassay [6], immunochromatography [7], piezoelectric methods [8], some molecular biological methods [9] and spectroscopic methods [10]. These methods have enriched the detection systems of abrin, but more or less possess several weak points such as complicated operation, long detection time or low sensitivity. As a rising technique in recent years, electrochemiluminescence (electrogenerated chemiluminescence (ECL)) sensing method has also been used for testing abrin, and obtained a good result with the LOD of 0.1–0.5 ng/mL [5].

The ECL sensor has extensive advantages, such as high sensitivity, wide detection range, good controllability, fast response and strong anti-interference ability and so on, which make it especially suitable for the detection of trace and ultra-trace amount of complex substances [11,12,13,14,15,16,17,18,19]. However, traditional ECL detection equipment based on magnetic separation technology needs to integrate some necessary components including discrete three-electrode system (working electrode, counter electrode and reference electrode), ultra weak photoelectric detection system, focusing lens system, magnetic separation and enrichment system and micro channel sampling and cleaning system into a unified ECL detection pool, which brings it some defects of high price, bulk and weight, inconvenient cleaning and so on. All of these have hindered the popularization and application of ECL sensor to a certain extent. To solve these problems and achieve in-situ, point-of-care testing, many novel designs have been proposed such as Lab-on-a-paper immunodevices [20] and microfluidic platforms [19]. In these simplified and efficient devices, a screen-printed electrode (SPE) often serves as an important, or indispensable, component.

SPE is prepared by screen printed technology. It can be produced in batches with a low price and it is miniature and portable with a good electrochemical performance [21,22,23,24,25]. Meanwhile, the SPE can be freely loaded and disassembled, bringing great convenience to the surface cleaning and modification of electrode, and the background interference caused by the inadequate cleaning of the traditional ECL reaction pool can be avoided. SPE highly integrates the three discrete electrodes. Used as a micro reaction pool, it can promote the miniaturization of ECL sensor and then enable environmental pollutants, biological warfare agents to be measured in situ. The portable ECL sensor based on SPE has broad application prospects in developing miniaturized, integrated and intelligent field inspection equipment.

In this study, the SPE is introduced into biotoxin detection by electrochemiluminescence immunoassay. Taking the virulent abrin as the target, and fully combining the advantages of SPE and magnetic separation immunoassay, we developed a portable ECL sensing system and establish a new method of ECL detection of biotoxin with high sensitivity and simplified operation, which can provide technical basis and reference for the outdoor environmental monitoring, food hygiene inspection, anti-bioterrorism and so on.

## 2. Materials and Methods

### 2.1. Reagents and Instruments

Ru(bpy)_3_^2+^-NHS ester, Biotin-NHS ester and DMF (*N*,*N*-Dimethylformamide) were purchased from Sigma-Aldrich (Munich, Germany). M-280 Streptavidin coated magnetic microspheres was purchased from Invitrogen Life Technologies (Oslo, Norway). Bovine serum albumin (BSA) was purchased from Shanghai Sinopharm Group Co., Ltd. (Shanghai, China) and Human IgG was purchased from Beijing Biosynthrsis Biotechnology Co., Ltd. (Beijing, China). Ricin, abrin, pcAb and mcAb of abrin were all prepared in our lab. DMSO (Dimethyl Sulphoxide) was purchased from Beijing Xingjin chemical plant (Beijing, China). The Procell solution mainly containing TPA (Tripropylamine) was purchased from Beijing Biolot Diagnostics Co., Ltd. (Beijing, China) and the deionized water was used as the experimental water.

**Figure 1:**
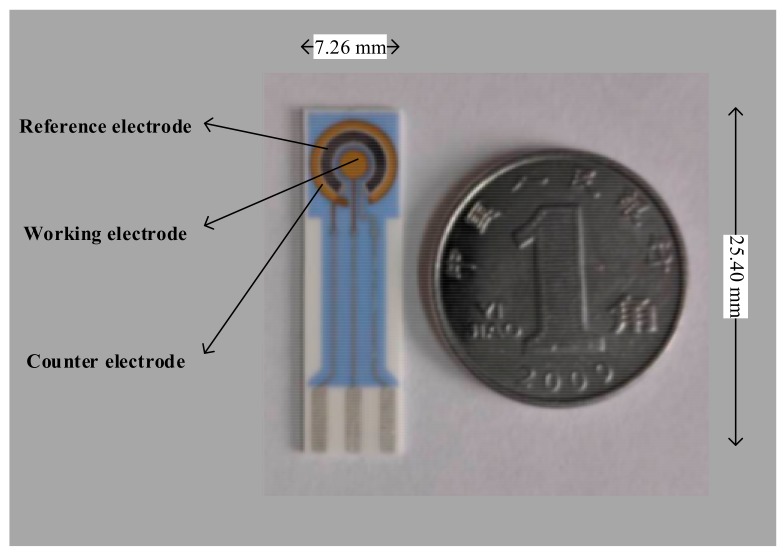
Size and real object of screen printed gold electrode (the right is the Chinese currency worth 0.1 Yuan, which is the minimum coin of China in circulation).

Screen-printed gold electrode (BVT Technologies, a.s., Czech; Figure 1) was the ECL reaction center. Portable ECL immunoassay sensor was jointly developed by our laboratory and Xi’an Remex Analysis Instrument Co. Ltd. (Xi’an, China). Samples were incubated by HS-3 vertical mixer (Scientz Biotechnology Co., Ltd., Ningbo, China). Preparation of biotinylated antibodies needed dialysis bag and RCT heating magnetic stirrer (IKA Company, Staufen, Germany). Magnetic separation operation was carried by Magnetic Separation Rack (Promega Company, Madison, WI, USA). A_280nm_ values and absorbance spectrum were determined on BioMATE 3S UV-Vis spectrophotometer (Thermo Fisher Scientific Inc., Waltham, MA, USA). Ru(bpy)_3_^2+^-labeled antibody was prepared by 1-15P centrifuge and VIVASPIN 500 ultrafiltration centrifuge tube (Thermo Fisher Scientific Inc., Waltham, MA, USA).

### 2.2. Design and Development of Portable ECL Sensing Platform

**Figure 2:**
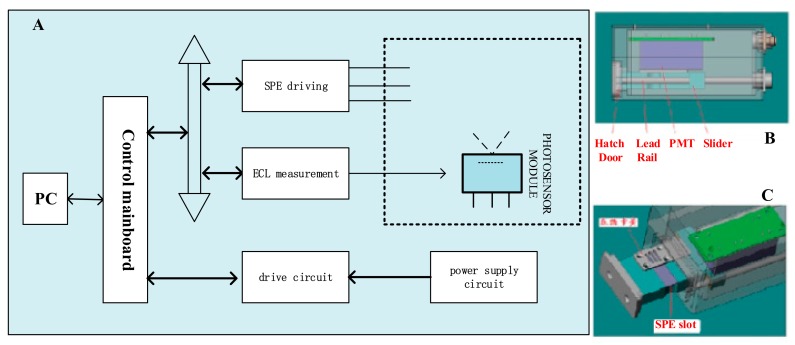
Overall structure of portable ECL detection platform: (**A**) the overall schematic diagram and main modules of the sensor including SPE, PMT, control mainboard, drive and power supply circuit; (**B**) the main mechanical structure of sensor including hatch door, lead rail, PMT and slider, and the whole shape is a small cube; and(**C**) a slot for clamping the SPE, which can pass in and out along the lead rail with the assistance of the spring.

The portable ECL sensing platform was jointly developed by our laboratory and Xi’an Remex Analysis Instrument Co. Ltd. (size: 18.2 cm × 14.0 cm × 5.2 cm, volume: 1.325 dm^3^, mass: 1.45 kg). In the design of this platform, the key point is to combine the photomultiplier tube (PMT) with SPE together organically, to solve the conundrum of integrated design of mechanical, circuit control, reaction driving, data acquisition problems and so on. Figure 2 indicates the general structure of portable ECL sensor (Figure 2A,B) and SPE clamping mechanism (Figure 2C). SPE and the clamping mechanism are supported by lead rail, they can move along the rail to the outside of the container with the assistance of the spring; The clamping mechanism is located at the hatch where are looped grooves which can attenuate natural light; PMT is fixed above the electrode surface. When the electrode and the clamping mechanism are completely moved into the container, the PMT’s light window is perfectly aligned with the luminous region on the electrode, enabling the sensor to collect signals with high efficiency.

There are two work modes for the portable ECL sensor: “independent work mode” and “online working mode”. The “independent work mode” means the sensor could perform a site survey in the absence of vehicle power (or electric supply) in the wild environment. It can detect, store information, display and print the result relying on the built-in rechargeable battery, SPE, internal storage, monitor and portable printer. When the sensor is in the “online work mode”, there is enough power supply (vehicle power or electric supply) and the sensor is connected to the computer. Utilizing the full-featured management software for detection and data exchange, more detailed test results could be output.

### 2.3. Experimental Method

Referring to methods from Liu [26] and Mu [27], this experiment mainly includes the following operations.

#### 2.3.1. Preparation of Capture Probe

pcAb was used to prepare magnetic microsphere capture probe. First, the pcAb needed to be biotinylated: 3.5 mL pcAb solution (1 mg/mL) was mixed with 0.5 mL DMF solution of activated biotin (biotin-NHS ester, 1 mg/mL), and stirred by magnetic force at room temperature for 3 h. Then, the reaction mixture was dialyzed overnight at 4 °C with 0.05 M PBS as the dialysis solution, and the purified antibody was stored at −20 °C for use.

Then, to immobilize the biotinylated antibody on the magnetic microspheres: 400 μL streptavidin coated magnetic microspheres was washed by 0.01 M PBS (PH = 7.4) adequately, and then 1 mL PBS and 200 μL biotinylated pcAb were mixed after magnetic separation, and incubated with moderate rotation at room temperature for 1 h. After the reaction was completed, the supernatant was obtained by magnetic force for absorbance detection to determine the binding amount of the capture antibody. Subsequently, the precipitate was suspended with 0.01 M PBS buffer and discard the supernatant by magnetic separation. This process was repeated five times to remove residual resistance and other impurities thoroughly. Finally, the precipitate was resuspended in 0.01 M PBS buffer and stored at 4 °C for use.

#### 2.3.2. Preparation of ECL Labeled Probe

Equal ratio DMSO and deionized water were used to prepare Ru(bpy)_3_^2+^-NHS ester solution (10^−3^ mol/L). Then, 200 μL was drawn to mix with 200 μL mcAb (2 mg/mL) and 600 μL carbonate buffer (0.05 M, PH = 9.6). The mixture was incubated for 12 h in the darkness, and then centrifuged at 8000 g for 10 min followed by cleaned with PBS for three times. Finally, the labeled antibody was resuspended in 0.01 M PBS buffer and stored at −20 °C for use.

#### 2.3.3. Determination of Abrin

**Figure 3:**
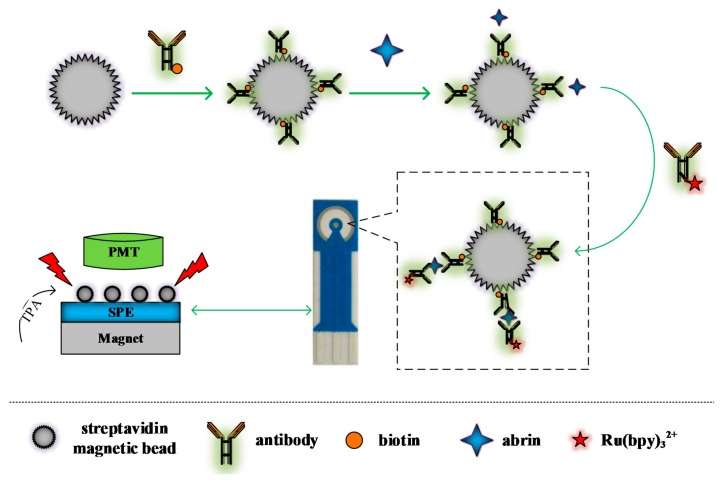
Flow diagram of detection of abrin.

Fifty microliters of capture probe and 50 μL abrin solution were mixed and incubated for 20 min at room temperature. After magnetic separation cleaning twice, 20 μL ECL labeled probe (100 μg/mL) and 80 μL 0.01 M PBS were added and incubated for 20 min [22,26]. Then, the mixture was cleaned five times, and 50 μL Procell solution was added into the precipitate for ECL detection. Five microliters of magnetic microspheres were absorbed and applied evenly to the surface of screen-printed gold electrode each time, and the ECL intensity was observed by cyclic voltammetry. The test process is illustrated in Figure 3.

## 3. Results and Discussion

### 3.1. Parameter Optimization of the Sensor

The core components of sensor are the SPE and the PMT, so several related factors including the additive amount of sample on the electrode surface and the multiplier series of PMT are likely to affect the detection results. In addition, pH value of sample and some other factors may also make a difference to detection effect. Therefore, optimizing the related factors or parameters will improve the sensitivity and accuracy efficaciously before experiment.

#### 3.1.1. Additive Amount of Sample

**Figure 4:**
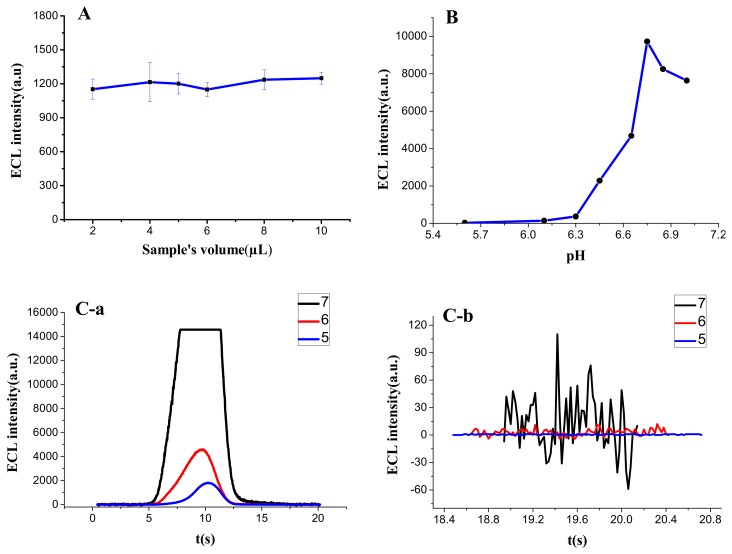
The effect of different factors on ECL intensity: (**A**) the effect of additive amount of sample on ECL intensity; (**B**) the effect of sample’s pH on ECL intensity; (**C**) the ECL intensity (**C-a**) and noise (**C-b**) of sample in different multiplier series of PMT.

While traditional ECL sensor detects the dynamic liquid sample with a peristaltic pump, the portable sensor could complete static analysis with only a drop of determinand on the surface of SPE. By comparison, the latter can reduce the sample demand and simplify the operation process. The diameter of three electrodes of SPE is 6 mm. To cover these electrodes completely without overflow, the amount of liquid drop should be between 2 μL and 10 μL. Within this range, the RSD of different groups (2 μL, 4 μL, 5 μL, 6 μL, 8 μL, and 10 μL) was only 3.5%, so the amount had no significant influence on the result of ECL detection (Figure 4A). Therefore, this experiment chose an intermediate value of 5 μL as the fixed quantity, which could not only increase the number of tests of one sample appropriately, but also prevent the rapid evaporation of Procell solution, resulting in a big change of detection value in a short time.

#### 3.1.2. pH of Sample

The ECL reaction principle of Ru(bpy)_3_^2+^-TPA system [28] is referred to as follows:

Ru(bpy)_3_^2+^ − e^−^ → Ru(bpy)_3_^3+^ (1)

TPA − e^−^ → [TPA^·^]^+^ → TPA^·^ + H^+^ (2)

Ru(bpy)_3_^3+^ + TPA → Ru(bpy)_3_^2+^* + products (3)

Ru(bpy)_3_^2+^* → Ru(bpy)_3_^2+^ + hv (4)

It can be found from Equation (2) that the concentration of H^+^ had great influence on the reaction. To explore the actual effect on the detection, the pH of Ru(bpy)_3_^2+^-NHS ester (1 × 10^−7^ M) prepared by Procell solution was adjusted by HCl or Na_2_CO_3_ ( note that TPA is an amine, thus the pH value cannot be too high), and then used for ECL test. The results (Figure 4B) indicated that the ECL intensity increased significantly with the increase of pH when the value was above 6, achieved the maximum level at pH = 6.75, and then gradually decreased. Therefore, Procell solution with pH value of 6.75 was used as luminous detection liquid in this experiment.

#### 3.1.3. Multiplier Series of PMT

Corresponding to the dynode, the multiplier series of PMT affects the sensing effect mainly from two aspects: As the series increased, the detection value increased in a geometrical progression (Figure 4C-a) while the noise also amplified proportionally (Figure 4C-b). When the amplification series was five, the noise was 1–2, but the sensitivity was low; when it was seven, the noise increased to 100–150, and the measured value was out of range; and when the multiplier series was set as six, the signal responded well with relatively low noise (10–15). Thus, after comprehensive comparison, the fixed multiplier series was selected as six.

### 3.2. The Characterization of ECL Capture Probe

**Figure 5:**
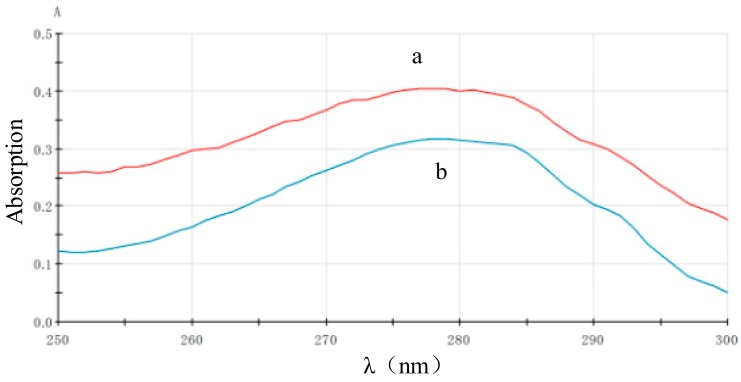
Absorbance spectrum of pcAb solution: before (a); and after (b) binding with magnet microspheres.

The magnetic microspheres contain a great quantity of streptavidin, which can stably combine with biotinylated protein. To confirm that pcAb had been combined with magnetic microspheres sufficiently, 400 μL biotinylated antibody solution (containing 100 μg pcAb, excess) and 200 μL magnetic microsphere (10 mg/mL) were mixed for coupling reaction, and the absorbance of pcAb solution at 280 nm was tested before and after reaction (A_(pro)_ and A_(post)_ respectively stood for the absorbance value at 280 nm before and after pcAb was bound). Figure 5 is an absorbance scanning spectrum between 250 nm and 300 nm, and A_(pro)_ = 0.400 and A_(post)_ = 0.315, so a result can be approximately calculated that the maximum amount of antibody fixed on magnetic microsphere was 111 μg/mL by the formula (A_(pro)_ − A_(post)_)/A_(post)_ × 100%, similar to the theoretical value stated by the manufacturer (about 100 μg/mL). This also indicated that the capture probe had been successfully prepared.

### 3.3. The Characterization of ECL Labeled Probe

#### 3.3.1. Characterized by the UV-Vis Absorption Spectrum

**Figure 6:**
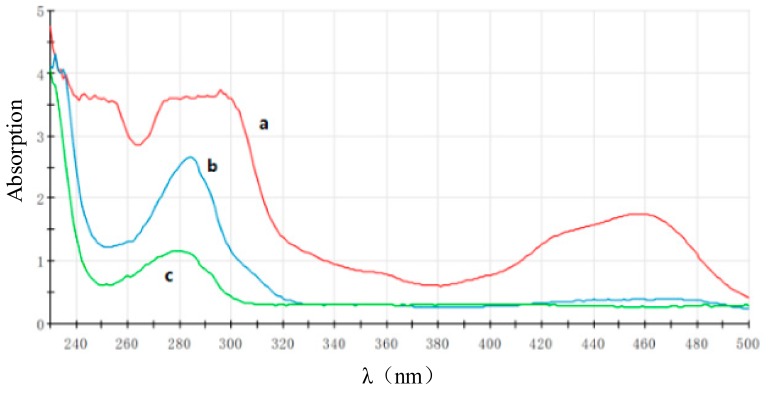
Absorbance of mcAb before (b) and after (c) labeled by Ru(bpy)_3_^2+^-NHS (a).

The UV-Vis absorption spectrum (Figure 6) shows there were three characteristic peaks of Ru(bpy)_3_^2+^-NHS ester (Figure 6, Curve a), located at 245 nm [Ru(II) to bpy ligand, dπ → π* transition], 287 nm [ligand centered, π → π* transition], and 457 nm [Ru(II) to bpy ligand, dπ → π* transition], respectively [26]. mcAb had only the characteristic absorption peak of protein at 280 nm (Figure 6, Curve c). The Ru(bpy)_3_^2+^-labeled mcAb is shown in Figure 6 as Curve b, and. As can be seen, there was an absorption peak at 280–287 nm (about 284 nm) with a medium height between mcAb and Ru(bpy)_3_^2+^-NHS ester, which could prove the ECL labeled probe has been prepared successfully. 

#### 3.3.2. Characterized by ECL Scanning

**Figure 7:**
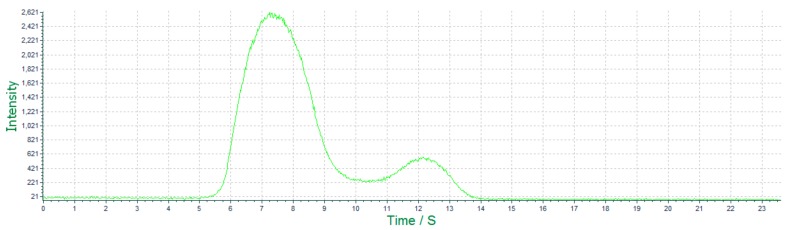
The real-time ECL scanning image of the labeled probe.

Figure 7 is the ECL cyclic voltammetric scanning image of the Ru(bpy)_3_^2+^-labeled probe, which reached the peak at about 1 V, and the spectrum’s characteristic was the same as that of Ru(bpy)_3_^2+^-NHS ester. It was a typical ECL scanning spectrum of materials containing the [Ru(bpy)_3_^2+^].

### 3.4. The Performance of the Portable ECL Sensor

#### 3.4.1. Limit of Detection (LOD) and Quantitative Range

**Figure 8:**
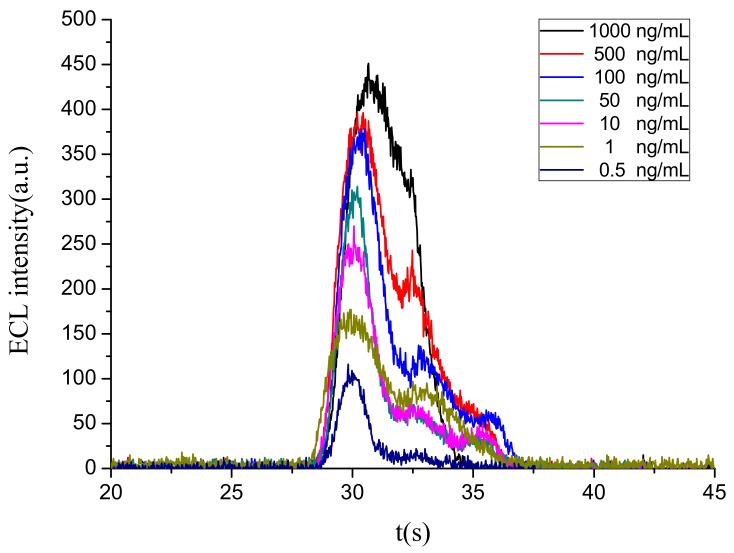
The ECL spectra for the abrin detection at different concentrations.

**Figure 9:**
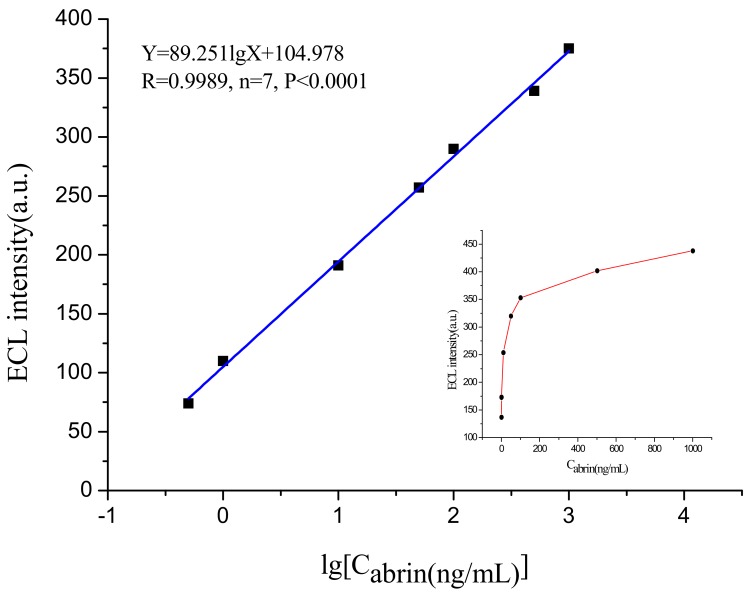
The relationship between ECL intensity and the concentration of abrin.

The established portable ECL sensor was used to test the magnetic microspheres complex binding with different concentrations of abrin standard substance and obtain the corresponding ECL intensity values. The integrated ECL spectra for the abrin detection at different concentrations is illustrated in Figure 8. Each concentration was tested at least four times to obtain an average ECL intensity value and the baseline value (63, value of blank control) needed to be deducted. Figure 9 shows the relationship between the ECL intensity and the abrin concentration. It can be noted that ECL intensity increased steadily with the increasing of abrin concentration. When the concentration exceeded 1000 ng/mL, the intensity no longer increased and reached the saturation state. Within the range 0.5–1000 ng/mL, ECL intensity (Y) and the logarithm of abrin concentration (X) showed a good linear relationship, and the regression equation was Y = 89.251lgX + 104.978 (R = 0.9989, *n* = 7, *p* < 0.0001), the LOD at the signal-to-noise ratio of 3 was 0.1 ng/mL. Compared with the piezoelectric immunoassay established by Mu [8], sensitivity of the portable ECL biosensor had been increased by more than 100-fold. Although this device was highly integrated and simplified, a similar result to the traditional ECL sensor [5,26] was still obtained.

#### 3.4.2. Reproducibility

In the quantitative detection range, 11 parallel determinations were performed on the concentration of 1 ng/mL, and the ECL intensity was 173 ± 17 with the RSD of 9.9%. In addition, the other six groups within the range were repeated four times, and the corresponding RSD values were, respectively, 16.5%, 8.3%, 10.2%, 4.7%, 3.6%, and 15.8%. The overall deviation was slight, and this portable sensor exhibited good reproducibility.

#### 3.4.3. Specificity

With 0.01 M PBS as the blank control, the ECL responses of 1 mg/L bovine serum albumin (BSA), ricin and human IgG were measured, and the response values were all at baseline (Table 1). It is worth noting that the spatial structure, toxic mechanism and clinical diagnostic features of ricin are very similar to those of abrin [1], which could also further prove that the detection of target protein by the sensor is highly specific. Dual specificity recognition with pcAb as the capture probe and mcAb as the luminescent probe, combined with the magnetic separation cleaning, ensured the high specificity of the sensing process.

#### 3.4.4. Detection of Spiked Samples

The practical value of the sensor can be assessed by testing the spiked samples. Milk, honey and soil samples (organic substance content > 5% or fat content > 30%) containing 100 ng/mL abrin were detected, respectively, and the measured values were calculated by regression equation (Table 2). The recovery rates of different samples were between 89% and 101%, meaning the portable sensor had an excellent application prospect in the detection of real samples.

## 4. Conclusions

The SPE has several typical characteristics of simple preparation, low cost, miniaturized and good electrochemical performance. Taking SPE as the reaction center, the authors built a portable ECL sensing platform, possessing some outstanding features such as simplified operation, miniaturization and portability. In this experiment, based on the portable sensor and magnetic separation immunoassay technique, a specific sandwich model was built and a successful detection of abrin was finally achieved. The LOD was 0.1 ng/mL and the quantitative range was 0.5–1000 ng/mL. The portable sensor exhibited high sensitivity, good reproducibility and excellent specificity, achieving a fine result better than the conventional ECL sensor. Subsequently, based on the feature that SPE is easy for surface treatment, nanophase materials can be used to modify SPE to improve the electrochemical performance and ECL reaction efficiency, thus greatly improving detection sensitivity. Then, the portable ECL sensor would have greater potential for application in the fields of physiological and pathological examination, environmental pollution monitoring and biological terrorism prevention.

## Figures and Tables

**Table 1:** Determination of substrate specificity (*n* = 3).

Substrate	conc. (ng/mL)	ECL Intensity	RSD (%)	Deviation (%)
BSA	1000	63.7	9.6	1.6
ricin	1000	67.7	6.7	8.0
Hu-IgG	1000	65	10.1	3.7
PBS(blank)	-	62.7	10.3	-

**Table 2:** Determination of abrin in spiked samples (*n* = 3).

Sample	Added (ng/mL)	Found (ng/mL)	Recovery (%)	RSD (%)
milk	100	97.7	97.7	12.7
honey	100	100.65	100.7	10.1
soil	100	89.1	89.1	9.2
